# Immune checkpoint inhibition mediated with liposomal nanomedicine for cancer therapy

**DOI:** 10.1186/s40779-023-00455-x

**Published:** 2023-04-28

**Authors:** Guang-Long Ma, Wei-Feng Lin

**Affiliations:** 1 Faculty of Medicine, Centre for Cancer Immunology, University of Southampton, Southampton, SO16 6YD UK; 2 Department of Molecular Chemistry and Materials Science, Weizmann Institute of Science, 76100 Rehovot, Israel; 3 Key Laboratory of Bio-Inspired Smart Interfacial Science and Technology of Ministry of Education, School of Chemistry, Beihang University, Beijing, 100191 China

**Keywords:** Liposome, Exosome, Immune checkpoint blockade (ICB)

## Abstract

Immune checkpoint blockade (ICB) therapy for cancer has achieved great success both in clinical results and on the market. At the same time, success drives more attention from scientists to improve it. However, only a small portion of patients are responsive to this therapy, and it comes with a unique spectrum of side effects termed immune-related adverse events (irAEs). The use of nanotechnology could improve ICBs’ delivery to the tumor, assist them in penetrating deeper into tumor tissues and alleviate their irAEs. Liposomal nanomedicine has been investigated and used for decades, and is well-recognized as the most successful nano-drug delivery system. The successful combination of ICB with liposomal nanomedicine could help improve the efficacy of ICB therapy. In this review, we highlighted recent studies using liposomal nanomedicine (including new emerging exosomes and their inspired nano-vesicles) in associating ICB therapy.

## Background

Cancer has been one of the leading causes of death for decades, and though the fight against cancer has never stopped, an estimated 10 million cancer deaths occurred in 2020 [1]. Many immune checkpoint blockades (ICBs), like ipilimumab, nivolumab, pembrolizumab, atezolizuma, durvalumab, and avelumab, have been approved by the Food and Drug Administration (FDA) for the treatment of cancer [2]. For example, pembrolizumab (Keytruda), the first anti-programmed cell death protein 1 (PD-1) agent approved by FDA, can bind to PD-1 on T cells to block its interaction with programmed cell death ligand 1 (PD-L1). Because PD-L1 is up-regulated in certain types of tumor, and when it is bound to PD-1, as an immune checkpoint, it inhibits the immune response of cytotoxic T cells. Thus, blocking the PD-1/PD-L1 pathway could restore the immune response [3–5]. However, traditional ICBs are usually monoclonal antibodies (mAbs), which have some drawbacks such as insufficient tumor penetration, inactivation, elimination due to cleavage by protease in vivo [6–10], and immune-related adverse events (irAEs) [2, 11]. Deveuve et al. [12] studied the cleavage of human immunoglobulin G (IgG)1 (trastuzumab, rituximab, cetuximab, infliximab, and ipilimumab), IgG2 (panitumumab), and IgG4 (nivolumab and pembrolizumab) structure based therapeutic mAbs in the presence of matrix metalloproteinase (MMP)-12 and immunoglobulin-degrading enzyme from *Streptococcus pyogenes*. Their results showed that IgG1 and IgG4 formats are sensitive to MMP-12 and immunoglobulin-degrading enzyme from *Streptococcus pyogenes*. The most common adverse events include colitis, diarrhea, dermatitis, hypophysitis, thyroiditis, and hepatitis [13–17]. Approximately 12% of patients on nivolumab monotherapy and 43% of patients on ipilimumab plus nivolumab faced treatment discontinuation due to adverse effects [15]. Those adverse events can also be life‐threatening. In a report, 613 of the 19,217 registered patients died as a consequence of treatment with immune checkpoint inhibitors. Toxicity‐related fatality rates were 0.36% for anti‐PD‐1, 0.38% for anti‐PD‐L1, 1.08% for anti‐cytotoxic T-lymphocyte-associated antigen 4 (CTLA-4), and 1.23% for PD‐1/PD‐L1 plus CTLA-4 [17]. Also, resistance to treatment is a big challenge. Up to 50% of PD-L1 positive patients show resistance or relapse post-ICB treatment [18–20]. Liposomal drug delivery systems have been successful in improving the therapeutic efficacy in cancer treatment [21–23]. Combining ICB and the advantages of liposomal drug delivery systems would potentially improve its therapeutic efficacy. In this review, we focus on studies that ICBs are encapsulated into/coated onto a liposomal delivery system, which will show its benefits directly compared to free ICB in the past 5 years. In addition, exosomes and exosome-inspired nanovesicles, new emerging drug delivery systems, which are composed of lipids, were also reviewed when combined with immune checkpoints blocking therapies.

## Liposomal nanomedicine and immune checkpoint in cancer therapy

### Liposomal nanomedicine in cancer therapy

**Fig. 1:**
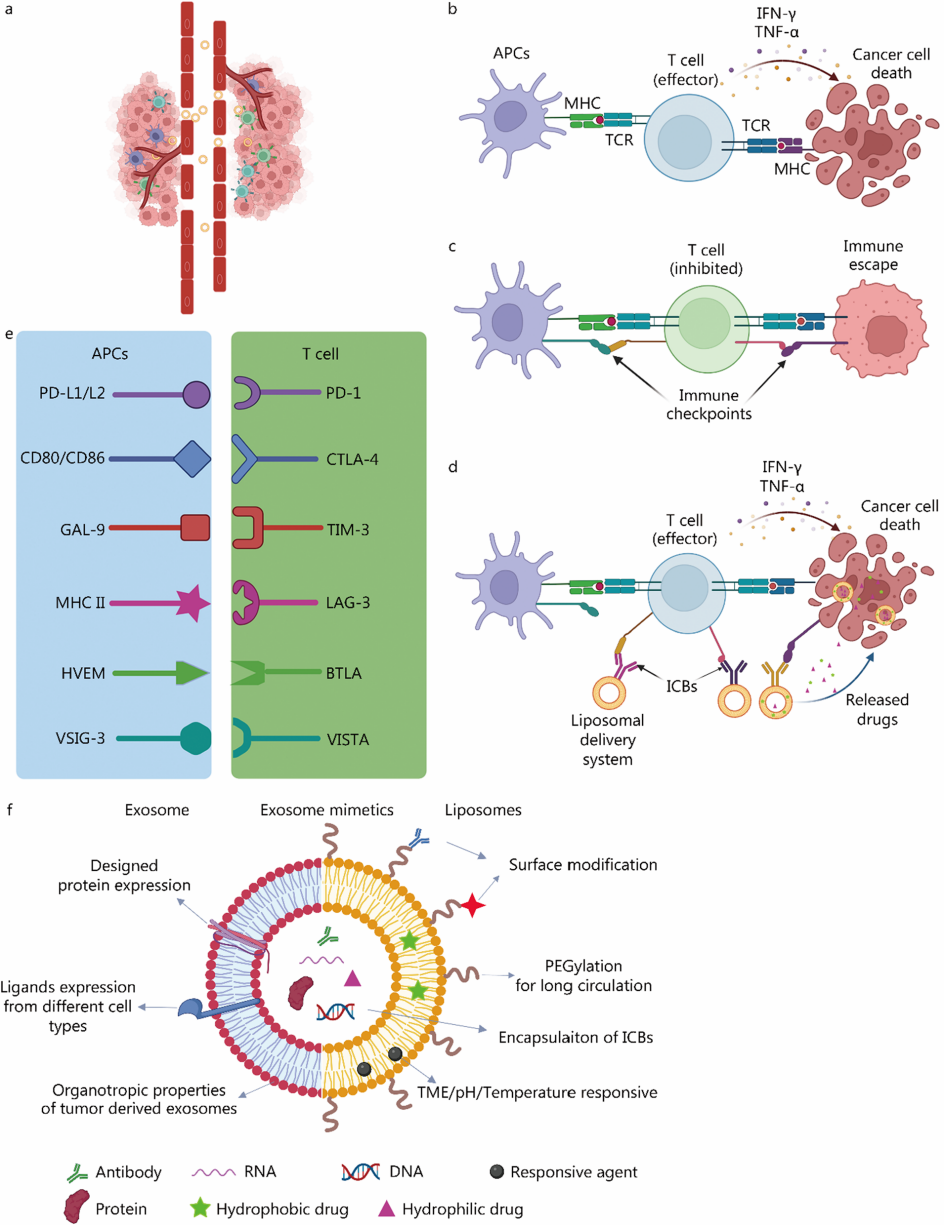
Summary of liposomal drug delivery system and immune checkpoint blockades. **a** EPR effect associated with liposomal drug delivery, liposomes with a diameter between 10 and 200 nm would preferably accumulate at the tumor site. **b** The process of T cell activated by APCs, and T cells led to cancer cell death. **c** T cells are inhibited by immune checkpoints, which leads to tumor immune escape. **d** ICB-modified liposomes reactivated T cells, and the reactivated T cell, together with tumor locally released drugs, led to cancer cell death. **e** Immune checkpoints’ ligand and receptors. PD-1/programmed cell death ligand 1 or 2 (PD-L1/2), CTLA-4/CD80/CD86, TIM-3/galectin-9 (GAL-9), lymphocyte-activation gene 3 (LAG-3)/major histocompatibility complex class II (MHC II), B and T lymphocyte attenuator (BTLA)/Herpes virus entry mediator (HVEM), V-domain Ig-containing suppressor of T-cell activation (VISTA)/V-set and immunoglobulin domain containing 3 (VSIG-3).** f** Representative structures of liposomal drug delivery system (liposomes, exosomes, and exosome mimetics). It was created with BioRender.com. EPR enhanced permeability and retention, APCs antigen-presenting cells, TCR T cell receptor, IFN interferon, TNF tumor necrosis factor, ICBs immune checkpoint blockades, PD-1 programmed cell death protein 1, CTLA-4 cytotoxic T-lymphocyte-associated antigen 4, TIM-3 T cell immunoglobulin domain and mucin domain 3, TME tumor microenvironment, DNA deoxyribonucleic acid, RNA ribonucleic acid

Conventional chemotherapeutic drugs usually have low aqueous solubility, poor pharmacokinetic parameters, and severe systemic toxicity due to the unbiased killing of cells. To reduce the drawbacks of these conventional drugs, nanomedicine was brought to the spot. Among them, liposomes, vesicular structures consisting of one or more phospholipid bilayers that formed impulsively in water, have attracted much attention due to their tunable nanometer size, facile loading for both hydrophilic and hydrophobic drugs, and high biocompatibility. Liposomes were first reported in the 1960s [24–26], and Doxil® was the first FDA-approved nano-drug in 1995 [a liposomal formulation of doxorubicin (DOX)] [21, 27]. Since then, many liposome-based nanomedicines have been developed and undergone clinical trials [21, 28]. Besides the improved solubility and bioavailability, they could also prevent the rapid clearance of drugs and improve the accumulation of drugs at the tumor site [29, 30]. One of the basic ideas behind the thriving of the nano-drug delivery system is the increased permeability of nanoparticles in solid tumors due to their aberrant vasculature, which is called the enhanced permeability and retention (EPR) effect (Fig. 1a). It is reported that nanoparticles with a diameter between 10 and 200 nm would have the most efficient therapeutic effect [31]. The extravasation mechanism could be both via the gaps between endothelial cells in the tumor vasculature and transcellular pathways by vesiculo-vacuolar organelles [32]. Though the EPR effect could improve the accumulation of encapsulated drugs at the tumor site, the encapsulation could also lead to decreased cytotoxicity [32]. Therefore, in addition to EPR resulted passive targeting, researchers have designed many tumor active targeting [33–37] and responsive [38–41] nano-drug delivery systems to enhance their therapeutic efficacy. For example, vascular endothelial growth factor was highly expressed on tumor cells’ surfaces, associated with their fast growth. Anti-vascular endothelial growth factor antibodies have been modified to the liposomes to improve the drug’s pharmacokinetics and tumor accumulation [42]. Zhou et al. [43] reported γ-glutamyl transpeptidase-responsive camptothecin–zwitterionic polymer conjugate that actively penetrates tumors via transcytosis to achieve enhanced anticancer efficacy. Such zwitterionic conjugate turns into positively charged polymers via cleaving with γ-glutamyl transpeptidase overexpressed on the cell membrane of luminal endothelial cells. This bio-responsive drug delivery system enables a uniform distribution throughout the tumor and significantly extends the survival rate of mice bearing pancreatic tumors. As a powerful delivery system, liposomes are not only successful in delivering chemotherapeutic drugs, but also are essential tools in developing new imaging modalities, theranostics, and vaccines, which have been extensively reviewed [22, 44–48].

### Immune checkpoint in cancer therapy

Though many drugs have entered clinical trials, and many of them have been approved by the FDA, many challenges remain to be tackled in curing cancers. The immunosuppressive tumor microenvironment (TME) is one of the trickiest challenges, posing a major barrier to cancer immunity. During tumor growth, numerous cancer antigens were released, which were then phagocytosed, processed, and presented by antigen-presenting cells (APCs) through the major histocompatibility complex. APCs, such as dendritic cells (DCs), migrate to draining lymph nodes, where the presented antigen can be recognized by T cells via T cell receptor, and initiate T cell activation (Fig. 1b) [49]. Following the T cell activation, T cells can also be regulated through antigen-independent co-inhibitory [CTLA-4, PD-1, V-domain Ig-containing suppressor of T-cell activation, and T cell immunoglobulin domain and mucin domain 3 (TIM-3), etc.] and co-stimulatory [CD28, inducible T cell co-stimulator (CD278), CD137 (41BB), and OX40, etc.] signals [5]. The co-inhibitory signals protect the body from excessive immune response, and co-stimulatory signals enhance T cell activation [2]. The co-inhibitory ligands/receptors, known as immune checkpoints, play a crucial role in maintaining immune homeostasis, minimizing the possibility of autoimmune inflammation. However, tumors can escape immune attack by the upregulation of the immune inhibitory mechanism (Fig. 1c) [20, 50]. Tumors can utilize specific immune-checkpoint pathways to achieve immune resistance, particularly against tumor antigen-specific T cells. Scientists have managed to block the ligand-receptor interaction to enhance cancer therapies (Fig. 1d). Among the commonly studied immune checkpoints (Fig. 1e), CTLA-4/CD80/CD86 and PD-1/PD-L1 are studied the most.

### Liposomal nanomedicine mediated ICB

**Fig. 2:**
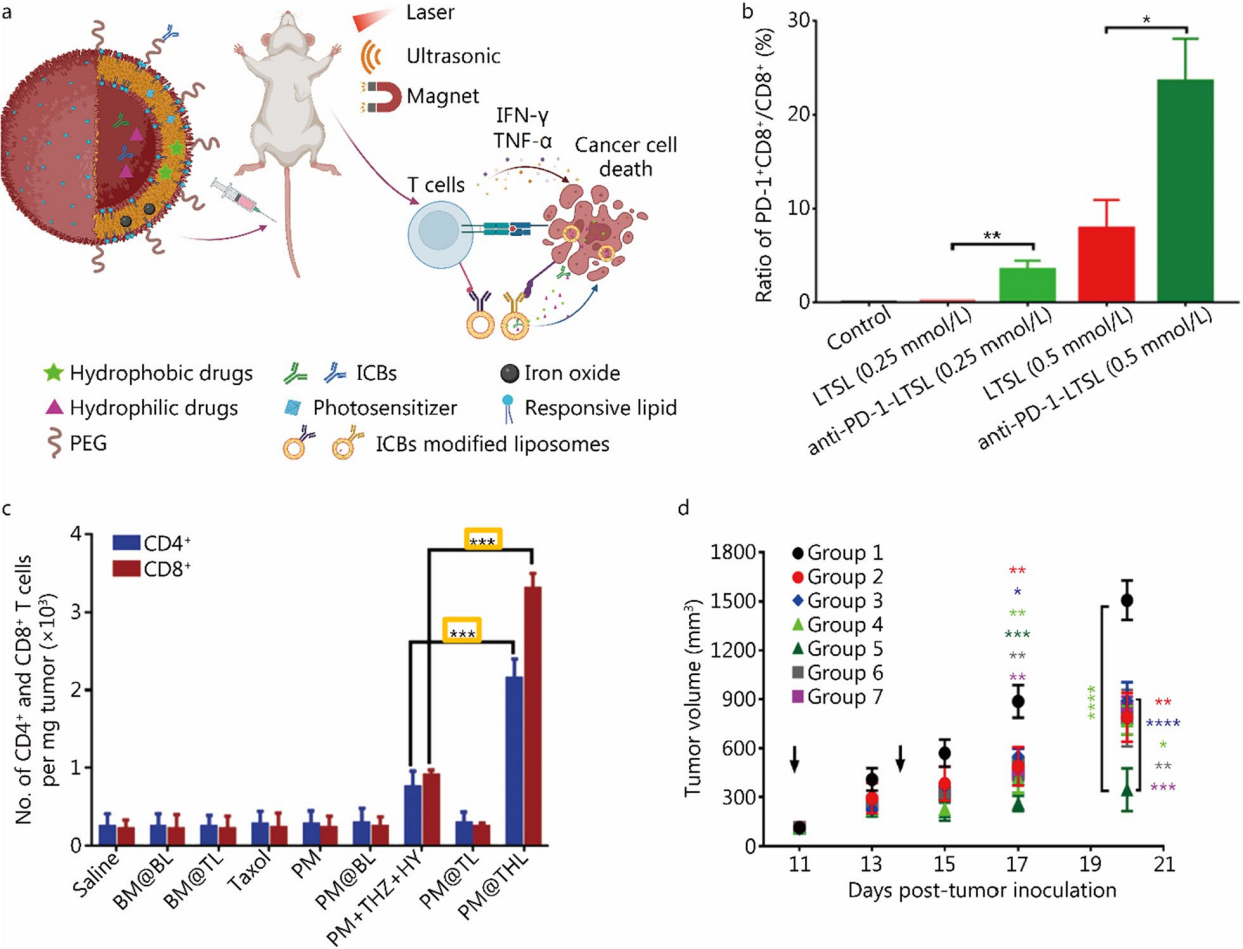
Liposome-associated immune checkpoint inhibition.** a** Illustration of multipurpose liposome delivery. **b** Anti-PD-1 modified liposome’s binding affinity to CD8^+^ T cells. Statistical analysis was performed by un-paired two-tail Student’s *t*-test, differences were considered significant at *P* < 0.05. **c** The increased tumor infiltrated CD4^+^ and CD8^+^ T cells in treatment with liposomal delivery associated ICB{BM@BL: blank micelle (BM) loaded hybrid liposome; BM@TL: BM/thioridazine (THZ)-loaded hybrid liposome; Taxol: commercial injection of paclitaxel (PTX); PM: PTX loaded poleyethylene glycol-block-poly[(1,4-butanediol)-diacrylate-β-N,N-diisopropylethylenediamine] (PDB) micelle; PM@BL: PM loaded hybrid liposome; PM + THZ + HY: PM together with free THZ and free PD-1/PD-L1 inhibitor HY19991 (HY); PM@TL: PM/THZ-loaded hybrid liposome; PM@THL: PM/THX/HY-loaded hybrid liposome. 4 mg/kg PTX, 16 mg/kg THZ, 4 mg/kg HY}. Statistical analysis was performed by one-way ANOVA and corrected by Bonferroni test for multiple comparison. **d** Growth curves of CT26 tumor inoculated subcutaneously in BALB/c mice and intravenously injected with PBS (Group 1, control, black dots), free DOX (Group 2, 2 mg/kg, red dots), anti-PD1 mAb (Group 3, 2.5 mg/kg, blue diamonds), mLTSL (DOX) (Group 4, DOX: 2 mg/kg, Fe: 3 mg/kg, light green triangles), mLTSL (DOX) + anti-PD1-LTSL (Group 5, DOX: 2 mg/kg, Fe: 3 mg/kg, anti-PD1 mAb: 2.5 mg/kg, dark green triangles), LTSL (DOX) (Group 6, DOX: 2 mg/kg, grey squares), and LTSL (DOX) + anti-PD1-LTSL (Group 7, DOX: 2 mg/kg, anti-PD1 mAb: 2.5 mg/kg, purple squares). Statistical analysis was performed by one-way ANOVA. ^*^P < 0.05, ^**^P < 0.01, ^***^P < 0.001, ^****^P < 0.0001. **a** was created with BioRender.com. **b** and **d** are adapted from ref. [75], published by Elsevier. **c** is adapted from ref. [90], published by Wiley. PEG polyethylene glycol, ICBs immune checkpoint blockades, IFN interferon, TNF tumor necrosis factor, LTSL low temperature-sensitive liposomes, anti-PD-1 anti-programmed cell death protein 1

Since liposomal nanomedicine has been successful in drug delivery, it would be beneficial to take advantage of the liposomal delivery system to improve the efficacy of ICBs (Fig. 1f). Liposomes with polyethylene glycol (PEG)ylation could shield them from reticuloendothelial system clearance, therefore having a longer circulation time. Formulated with TME-responsive lipids, such as pH, temperature, and redox, liposomes could give a burst release of payloads and minimize systemic toxicity. ICBs can usually be either encapsulated in the core of liposomes or modified onto the surface, along with other agents like a photosensitizer, and iron oxide, to have a combinatory therapy with external stimuli (Fig. 2a). The encapsulated ICBs can be protected from proteolytic cleavage, and surface modified ICB was also proved to maintain their binding affinity (Fig. 2b). Also, ICB associated with liposomal delivery could induce a better effective T cells tumor filtration and tumor inhibition compared to free ICB (Fig. 2c, d). In a recent study, CD25 antibody-modified pH-sensitive liposomes were used to transmigrate the endothelial barrier, infiltrate the TME, and release the encapsulated drugs (including ICBs) [51]. Also, a liposomal delivery system would allow multiple ICBs to be delivered simultaneously for combinatorial therapies [52].

There are also reports about remodeling the TME with a liposomal drug delivery system to sensitize tumors to checkpoint inhibitors, combined with the administration of free ICBs to achieve a better therapeutic effect [53–56]. For example, in treatment metastasis, cancer cells that spread to surrounding tissues from the original tumor and the major cause of treatment failure and tumor recurrence, Huang et al. [56] first loaded indocyanine green as a photothermal agent into liposome for photothermal therapy (PTT). PTT alone can efficiently eradicate the primary tumor, meanwhile having minimal effect on the inhibition of distant tumors, which is caused by the compensatory upregulation of immune checkpoints after PTT. When PTT was combined with free anti-PD-1 and anti-TIM-3 antibodies administration, the growth of distant tumor was successfully inhibited while the primary tumor was cleared. These are also good strategies for improving therapeutic efficacy. Cremolini et al. [57] and Lahori et al. [58] reviewed enhanced ICBs, either delivered by or in combination with different nanocarriers. Gu et al. [49] reviewed the liposome systems developed for cancer immunotherapy, in which many immunomodulatory molecules, like stimulatory molecules and ICBs, are discussed.

## Liposomal nanomedicine mediated immune checkpoint inhibition

### Liposomal nanomedicine mediated CTLA-4 blockade

CTLA-4 is a member of the CD28-B7 immunoglobulin superfamily and expresses on both activated T and regulatory T (Treg) cells. In the early stage of T cell activation, CTLA-4 is up-regulated, and it negatively regulates T cell activation by competing with the CD28 receptor for binding CD80/CD86 ligands on APCs. CTLA-4 has higher affinity and avidity compared to CD28 and leads to the inhibition of antigen presentation by APCs, T cell proliferation, and reduced cytokine secretion [2, 59, 60]. However, ipilimumab is the first and only CTLA-4 inhibitor approved by the FDA in 2011 for the treatment of melanoma (a type of skin cancer) [61]. Despite the rapid approval of anti-PD-1/PD-L1 ICB, anti-CTLA-4 ICB failed in multiple phase III clinical trials, and CTLA-4 monotherapy showed more irAEs [62]. As CTLA-4 is important in preventing autoimmunity, the unselective blockade of CTLA-4 could be the major cause of its related irAEs [60, 62].

To reduce CTLA-4 blockade’s irAEs, and improve its therapeutic efficacy, Nikpoor et al. [63] encapsulated CTLA-4 blocking antibodies into both PEGylated (PEG modified) and non-PEGylated liposomes. The CTLA-4 blocking antibody encapsulated liposomes had good encapsulation efficacy and stability. At the same time, the PEGylated one showed longer blood half-lives and tumor accumulation compared to non-PEGylated liposomes and free CTLA-4. Though no significant difference in tumor infiltrated lymphocytes between different groups was observed, the CTLA-4 blocking antibody encapsulated PEGylated liposomes group showed the highest CD8^+^ T cells, T effector to Treg ratio, the best tumor inhibition, and the highest survival rate in CT26 colon carcinoma tumor models. Later in the same group, Alimohammadi et al. [11] combined chemotherapy (Doxil) and immunotherapy (anti-CTLA-4 antibody, free or PEGylated liposome-encapsulated) in treatment of well-established B16 mouse melanoma model. In this study, they assessed the effect on tumor inhibition of injection sequence, which showed that administration of free anti-CTLA-4 antibodies before Doxil had a better response compared to reversed order or concomitant with Doxil. Comparing to free anti-CTLA-4 antibody + Doxil, CTLA-4 PEG-liposomes (modification of liposomes by covalent conjugation with PEG) + Doxil showed even better tumor inhibition and survival rate. These results indicated that encapsulating anti-CTLA-4 antibodies into liposomes has good potential for tumor treatment, and this may be a new strategy for anti-CTIL-4 antibody development. However, complete in vivo toxicity investigation and stability studies are needed for further development.

### Liposomal nanomedicine mediated PD-1/PD-L1 blockade

Similar to CTLA-4, PD-1 is also expressed in T cells, B cells, DCs, and natural killer cells in the process of T cell activation [64]. But different from CTLA-4, which mainly enhances the immunosuppressive activity of Treg cells during T cell priming and activation, the PD-1 checkpoint works on cytotoxic CD8^+^ T cells [65]. There are two ligands of PD-1, PD-L1, and PD-L2. PD-L1 is up-regulated on activated T cells, B cells, DCs, macrophages, other hematopoietic cells, and many tumor cells. PD-L2 is mainly up-regulated on activated T cells, B cells, and other tissue-derived immune cells [2, 61, 64]. The PD-1/PD-L1/PD-L2 pathway plays a vital role in preventing autoimmune disease. However, this would suppress the immunological function and lead to tumor immune escape [2]. As many studies have reported that PD-L1 is overexpressed on tumor cells, which leads to the inhibition of the cytotoxicity of T cells and therefore accelerates tumor progression [66]. The design of ICB to inhibit this signaling pathway attracted scientists’ attention. Many ICBs have been approved by FDA either by blocking PD-1 (nivolumab, pembrolizumab) or PD-L1 (atezolizumab, durvalumab, avelumab). The direct role of PD-L2 in cancer progression and immune-TME regulation is not as well studied as the role of PD-L1. To our knowledge, there is no FDA-approved inhibitor for PD-L2 yet.

#### PD-1/PD-L1 blockade with chemotherapy

Nonetheless, only a small portion of patients are responsive to this ICB. Therefore, it’s also essential to improve its therapeutic efficacy in combination with other techniques. ICB can be combined with chemotherapy using liposomal drug delivery systems. As liposomes were first introduced to improve the pharmacokinetics and safety of chemotherapy, these could also be applied to ICB. Merino et al. [67] prepared liposomes composed of lipids including anti-PD-L1 monovalent variable fragment (Fab’) conjugated 1,2-distearoyl-sn-glycero-3-phosphoethanolamine-N-[amino (polyethylene glycol)-2000] (DSPE-PEG2000). Then the liposomes were loaded with DOX (LPF). Both non-anti-PD-L1 liposome (LPD) and LPF showed slow release in 100% FBS at 37 °C (less than 10% in 1 h) which indicated its stability in the biological environment, and no morphology change up to 3 months in N-2-hydroxyethylpiperazine-N-2-ethane sulfonic acid saline (pH 6.7) at 4 °C. LPF showed faster uptake in a PD-L1 expressing cell line, and higher toxicity than conventional LPD. Though the authors reported no statistical CD8^+^ cells difference in tumor, they found the anti-PD-L1 modified liposomes promoted a significant increment of specific and active tumor-infiltrating T cells. LPF showed the best tumor inhibition among all the groups, including free DOX, LPD, and LPD^+^ free anti-PD-L1.

Irinotecan (IRI) is a type of chemotherapy that can block topoisomerase I, which is needed by cells for dividing and growing, and it can also induce immunogenic cell death (ICD) [68]. ICD is dying cells’ exposure to damage-associated molecular patterns in the TME, which stimulates the antitumor immune system [69, 70]. JQ1, a small molecule inhibitor that could competitively bind to bromodomain, has been reported to show anti-proliferative effects in many types of cancers, and also has been used as a PD-L1 suppressor [68, 71]. He et al. [68] designed an IRI and JQ1 co-delivery liposomal system (Lipo), combining chemotherapeutic efficacy with JQ1-based PD-L1 suppression. Of note, they also conjugated anti-PD-L1 antibodies to the surface of the liposomes (P-Lipo), and they claimed the modification is for targeting purpose, not for blocking the PD-1/PD-L1 pathway, as the amount of anti-PD-L1 is less than 10% of the effective dose. The data showed that chemotherapeutic IRI up-regulated PD-L1 expression in tumor cells, confirming the importance of combination treatment with a PD-L1 inhibitor. The population of interferon (IFN)-γ^+^CD8^+^ T cells in the tumor treated with P-Lipo is 13.6%, higher than saline (1.1%) or free JQ1 (3.6%). Furthermore, the population of Tregs in the tumor is decreased from 18.2% (saline), about 13% (free JQ1) to 6.8% (P-Lipo).

PD-L1 can recycle back to the cell membrane after internalization with mAbs binding, which will affect the T cell-mediated antitumor immunity [72]. Yang et al. [73] designed PD-L1 multivalent binding liposomes to bias the PD-L1 toward lysosomes for degradation instead of recycling endosomes, which will lead to the decrease of PD-L1 level. They conjugated anti-PD-L1 peptide to DSPE-PEG (αPD-L1-Lipo) and prepared liposomes with different ratios. 10 mol% PD-L1 binding peptide (10-PD-L1-Lipo) promoted PD-L1 multivalent binding on the tumor cell membrane and led to lysosomal degradation instead of endosomal recycling. This alone showed better tumor inhibition than free anti-PD-L1 antibody and free anti-PD-L1 peptide. They further synergized the system by loading DOX in liposomes for immunogenic chemotherapy and showed significantly enhanced antitumor efficacy and immune responses in colon tumor models.

#### PD-1/PD-L1 blockade with external stimuli-responsive liposomal nanomedicine

To improve its therapeutic efficacy even further, external stimuli are often applied to achieve better results. Local mild hyperthermia (HT) has been used to enhance tissue perfusion and local drug release in tumor treatment. ThermoDox^®^ (Celsion Corporation), a low temperature-sensitive liposomes (LTSL) formulation, has completed its phase III clinical study in combination with standardized radiofrequency ablation in primary liver cancer. However, some recent studies have shown that HT will up-regulate the PD-L1 expression on tumor cells, thereby making the TME immunosuppressive [74]. As a result, blocking the PD-1 on the surface of T cells while applying mild HT would also give a promising development. Based on this, Ma et al. [75] combined mild HT with anti-PD-1 ICB. They first embedded iron oxide into the bilayer of LTSL (mLTSL), then loaded with DOX [mLTSL (DOX)]. In the meantime, anti-PD-1 antibodies were conjugated to the surface of LTSL (anti-PD-1-LTSL), while maintaining their binding capacity to CD8^+^ T cells (Fig. 2b). DOX as an anticancer agent that induces ICD, was fast released from mLTSL (DOX) locally when applied with near-infrared (NIR) laser. The mild HT would also sensitize the tumor for immunotherapy. At the same time, infiltrated T cells with anti-PD-1-LTSL accumulated at the tumor site, leading to colon tumor inhibition. Their results showed a significantly higher level of IFN-γ in the serum and better tumor inhibition compared to free anti-PD-1 antibodies, which confirmed the advantage of using a liposomal delivery system. At the same time, the embedded iron oxide made this system a good platform for magnetic resonance imaging.

Though the ICB can reverse the immunosuppressive TME, the tumor infiltration of lymphocytes in many tumors is limited. To turn the immunologically “cold” tumors into “hot” and synergize with ICB, Huang et al. [74] co-loaded a photothermal agent (IR820) and an anti-PD-L1 antibody into a lipid mixture which will undergo a reversible gel-to-sol transition with the application of NIR laser. They successfully increased the level of matured DCs in inguinal lymph nodes, and CD8^+^ and CD4^+^ T cells infiltrated into 4T1 tumors. As expected, this led to significant tumor inhibition. In addition, it also inhibited the distal tumor’s growth and rechallenged lung metastasis. They also demonstrated the broad applicability of this system by investigating its B16F10 melanoma tumor inhibition, which also showed enhanced tumor inhibition and prolonged survival rate.

As mentioned above, ICD also plays an important role in cancer treatment [70]. However, the extensive tumor stroma and dense extracellular matrix limit ICD-inducing agents’ tumor penetration, and the immunosuppressive TME inhibits the immune system’s antitumor immunity [76]. Combining ICD and ICB with a liposomal drug delivery system could ideally solve the problem. Yu et al. [76] tried to combine ICB, immunogenic death, PTT, and tumor targeting at one go in a liposomal system. They integrated IR780 (photothermal agent), folic acid (FA) linked oxaliplatin (OXA) prodrug (tumor targeting + ICD), BMS-1 (PD-L1 inhibitor), and lipids to form thermosensitive liposomes using lipid film hydration method. The liposomes allow the tumor accumulation via the EPR effect, and upon NIR laser irradiation, OXA prodrug and BMS-1 were fast released in a few minutes. FOIB@Lip (including IR780, FA-OXA, and BMS-1) with laser irradiation showed better immunogenicity and tumor inhibition compared to FOIB@Lip without laser irradiation, indicating the importance of PTT in this system. The better tumor inhibition of FOIB@Lip with laser irradiation compared to FOI@Lip (including IR780, FA-OXA, but not BMS-1) with laser irradiation proved the importance of PD-L1 ICB.

Similar to using NIR laser as an external stimulus, ultrasound is also a good choice due to its deep penetration and non-invasiveness [77]. To achieve a high anti-PD-1 antibody loading, controllable drug [paclitaxel (PTX)] release, and precise optical imaging formulation, Li et al. [77] first used TiO_2_ shell (sonosensitizer) to encapsulate ZnGa_2_O_4_:Cr^3+^ (ZGO for luminescence imaging) and anti-PD-1, then this was loaded into the core of PTX loaded liposomes during the hydration process. Second, neutrophils (NEs) as the carriers were loaded with the prepared formulation, because NEs are believed to adhere to and migrate across endothelial vessels into the tumor site via an intercellular route. NE transportation enabled efficient blood–brain-barrier penetration of delivery vehicles for glioblastoma (a tumor of the central nervous system) treatment. Ultrasound-triggered local–regional chemotherapy and immunotherapy eradicated the primary tumor and inhibited the formation of metastasis, which led to a significant increase in survival without off-target systemic toxicity.

#### PD-1/PD-L1 blockade with tumor environment responsive liposomal nanomedicine

Besides the synergetic therapy of ICB and PTT/photodynamic therapy (PDT) (external stimuli), researchers also utilize ICB with chemotherapy in responsive liposomal drug delivery systems. As PD-1/PD-L1 is essential in preventing autoimmunity, improving the ICB accumulation at the tumor site is very important. The aberrant behavior of cancer cells could be advantageous to have a safer ICB therapy. The weak acidic microenvironment of tumors (pH 5.6–6.8) is a typical characteristic of malignant tumor cells. It is due to increased fermentative metabolism and insufficient blood perfusion, which is a target for intelligent cancer nano-theranostics [78–80]. Gu et al. [81] used anti-PD-L1 and docetaxel encapsulated pH-sensitive liposome (PDL) to synergize chemotherapy with ICB. Much faster drug release was obtained at acidic pH in vitro. PDL showed higher tumor cell apoptosis compared to a free combo of docetaxel and anti-PD-L1, as well as a significant delay of tumor growth. Such liposomes may modulate targeted delivery and active drug accumulation in tumor sites, and diminish unwanted adverse effects on normal organs. An elevated level of reactive oxygen species (ROS) has been observed in cancers for various reasons, such as increased metabolic activity, mitochondrial dysfunction, and increased cellular receptor signaling [82–84]. To overcome the low bioavailability and drug resistance of the hydrophobic drug PTX, Wang et al. [85] co-loaded BMS-202 (a small molecule that acts as a PD-1/PD-L1 inhibitor) and PTX derivative into a ROS-responsive liposome through a remote-loading method with a high drug loading. The ROS-responsive thioether bond in PTX-derivative allows a burst release of PTX in the tumor site without premature release, together with a sustained BMS-202 release to achieve a highly efficient chemo-immunotherapy. MMPs are a large family of zinc-dependent proteolytic enzymes that are important in the degradation of extracellular matrix, and more and more evidence has shown that they are related to the tumor invasion and metastasis [86, 87]. MMPs are often up-regulated and overexpressed in cancer, utilizing this could provide a localized controlled release in tumor tissues [88]. Zhang et al. [89] grafted synthetic PD-L1 peptide antagonists (P peptide) to mannose-modified liposomes through MMPs cleavable octapeptide. Afterward, the liposomes were coated with hyaluronic acid and loaded with oligodeoxynucleotides containing unmethylated cytosine and guanine motifs (to stimulate macrophages for continuous release of cytokines). In their study, P peptide grafted liposomes (monotherapy) showed more obvious tumor inhibition compared to non-P peptide grafted liposomes, and this provided a new way of investigating safer ICBs delivery. Though the combination of ICBs with chemotherapy could effectively kill cancer cells, cancer stem cells (CSCs) may still lead to recurrence and increased resistance in some circumstances [90–92]. In this case, anti-CSC treatment should also be included. Due to the multiple-agents co-loading capabilities of the liposomal drug delivery system, Lang et al. [90] reported a cocktail strategy of loading PTX, thioridazine (TDZ, anti-CSC agent), and HY19991 (HY, PD-1/PD-L1 inhibitor) into an enzyme/pH dual sensitive liposomal structured nanoparticles. They first prepared pH-responsive micelles loaded with PTX (named PMs), then PMs were co-encapsulated along with HY and TDZ, into MMP cleavable liposomes. The MMP in the tumor environment could lead to the release of PMs, HY, and TDZ. Then the released PMs, which have a particle size of around 50 nm, could penetrate cancer cells more efficiently than free PTX. The uptaken PMs would release their cargoes once they were endocytosed and transported to endosomes/lysosomes. This strategy showed more tumor accumulation, longer blood circulation, and effective T cell penetration into tumors (Fig. 2c) when compared to injecting the free agents. As a result, significantly improved tumor inhibition and decreased metastasis were observed.

#### PD-1/PD-L1 blockade with gene delivery liposomal nanomedicine

Gene delivery has rapidly emerged as a powerful tool in the treatment of cancers. Different from classic PD-1/PD-L1 antibodies or antagonists, knockout of either PD-1 or PD-L1 using gene delivery technology could also bring new insight into ICB therapy. Lu et al. [93] encapsulated clustered regularly interspaced short palindromic repeats/clustered regularly interspaced short palindromic repeats-associated protein 9 into liposome to specifically knockout PD-1 gene from T cells. Similarly, as CD47 and PD-L1 are critical innate and adaptive checkpoints, Lian et al. [94] designed high-epithelial cell adhesion molecule cancer cells targeting cationic liposome (LPP-P4-Ep) that contains si-CD47 and si-PD-L1, which could knockdown both CD47 and PD-L1 proteins. With the same idea, Barati et al. [95] prepared liposomes with PD-1 silencing small interfering RNA (siRNA) to enhance anti-tumor immune responses.

### Liposomal delivery system associated with other ICBs

Along with the extensive studies on CTLA-4 and PD-1/PD-L1 immune checkpoints, more and more immune checkpoints that can be blocked to associate with the therapeutic treatment of cancer have been found. Such as TIM-3 [2, 65, 96–99], lymphocyte-activation gene 3 [2, 65, 96–99], human endogenous retrovirus-H long terminal repeat-associating 2 [2], B7 homolog 3 protein [2, 65, 96], B7 homolog 4 protein [2, 96], V-domain Ig-containing suppressor of T-cell activation [97–100], B and T lymphocyte attenuator [101, 102], and CD37 [103]. But to our knowledge, there is no liposomal delivery system designed for these ICB yet. Besides these immune checkpoints, some other receptors or mediators can be targeted in the TME [49].

C-X-C chemokine receptor type 4 is a chemokine receptor, and its upregulation in tumor tissues (both on the cell surface and cytoplasm) is associated with increased immunosuppression in TME [104–106]. As the insufficient T cell infiltration in triple-negative breast cancer limited its response to normal ICB, Lu et al. [107] incorporated and modified plerixafor (AMD3100, a C-X-C chemokine receptor type 4 antagonist) into the aqueous core and on the surface of liposomal nanoparticles. Their results showed that liposomal-AMD3100 had higher CD3^+^ T cells and fewer Tregs infiltrated into 4T1 tumors than free AMD3100. Also, the data showed that liposomal-AMD3100 has more significant tumor-suppressive cytokines (INF-γ, IL-12a) upregulation and immunosuppressive cytokines [IL-10, transforming growth factor-β (TGF-β)] downregulation compared to free AMD3100.

Indoleamine-2.3-dioxygenase 1 (IDO1) is a cytosolic enzyme that catalyzes essential amino acid tryptophan to kynurenine, whose metabolites will lead to the suppression of T cells and are responsible for tumor immune escape. It is also associated with poor prognosis in various cancer [108–110]. To improve biocompatibility and tumor accumulation, Huang et al. [111] prepared a conjugate of protoporphyrin IX as a photosensitizer and NLG919 as an IDO1 inhibitor, and it was encapsulated into liposomes. The combined PDT and ICB achieved both primary and distant tumor inhibition. The results showed that combining PDT with ICB had better tumor inhibition than PDT alone and much better than ICB alone. Tumor-responsive liposomal delivery is always a good choice for reducing off-target toxicity. The high level of glutathione in tumors has been utilized to design a redox-active delivery system. Liu et al. [112] designed a redox-active liposome with a photosensitizer conjugated lipid with a reduction-sensitive link. This allowed the ROS generation of photodynamic triggered ICD, and along with the release of encapsulated IDO1 inhibitor, the further systemic antitumor immune response was augmented.

## Exosomes and exosome-inspired nanovesicles mediated ICBs

**Fig. 3:**
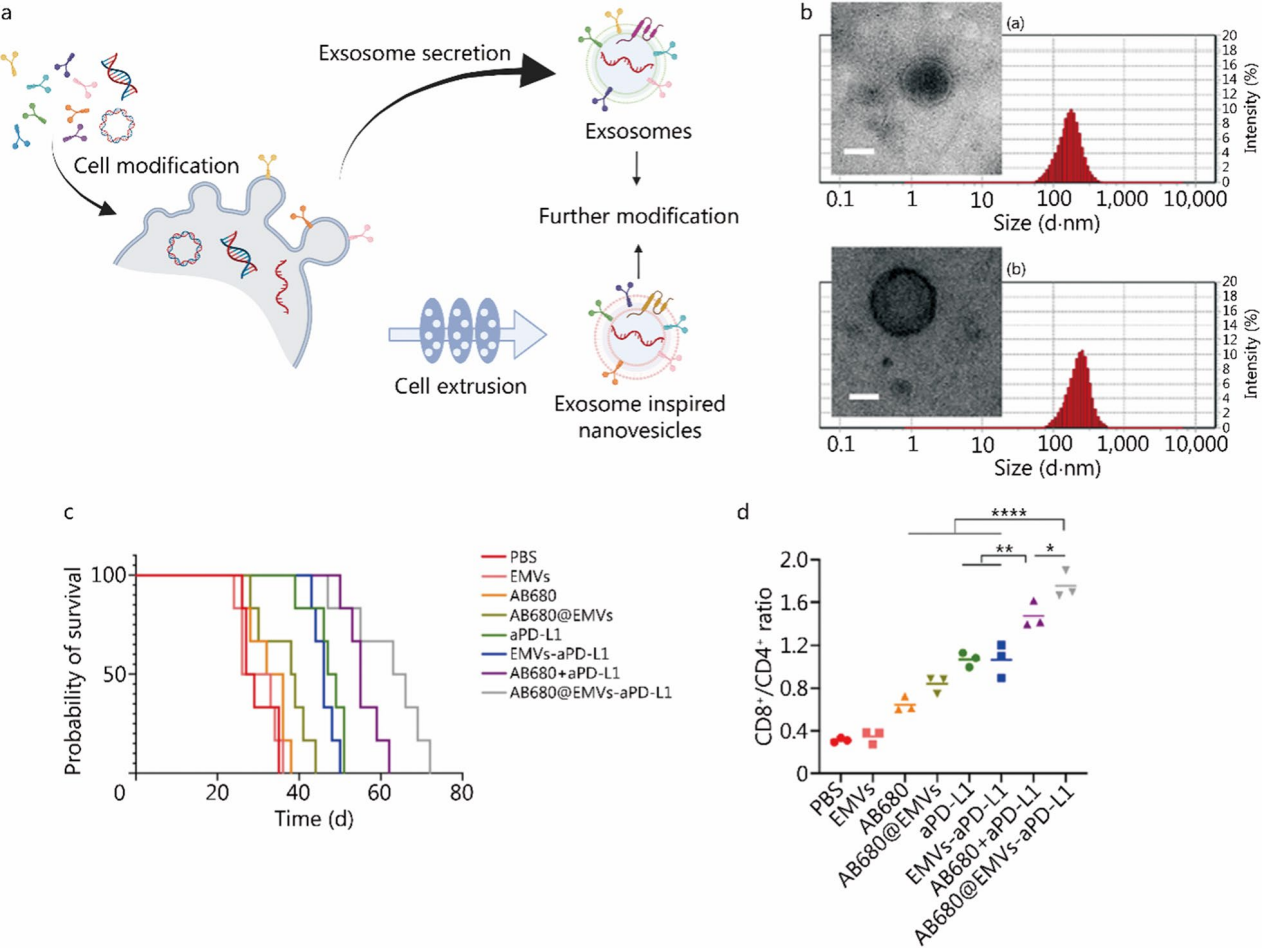
Exosome-inspired nanovesicles associated with immune checkpoint inhibition. **a** Schematic of ICB-modified exosomes and exosome-inspired nanovesicles. **b** Transmission electron microscopy images and size distributions of (a) exosome-inspired nanovesicles, and (b) ICB modified exosome inspired nanovesicles. **c** Median survival of mice treated with free ICBs or exosome inspired nanovesicles associated ICBs (PBS: Dulbecco’s phosphate-buffered saline; EMVs: exosome-mimetic nanovesicles; AB680: free CD73 inhibitor; AB680@EMVs: AB680 encapsulated EMVs; aPD-L1: free anti-PD-L1; EMVs-aPD-L1: EMVs conjugated with anti-PD-L1; AB680 + aPD-L1: free AB680 together with free anti-PD-L1; AB680@EMVs-aPD-L1: AB680 encapsulated EMVs conjugated with anti-PD-L1 on the surface). **d** Improved CD8^+^/CD4^+^ ratio in tumor tissues with treatment of exosome inspired nanovesicles associated ICBs. ^*^P < 0.05, ^**^P < 0.01, ^****^P < 0.0001. **a** was created with BioRender.com, **b–d** are adapted from ref. [129], published by American Chemical Society. ICB immune checkpoint blockade, EMVs exosome-mimetic nanovesicles

Exosomes are one of the main classes of extracellular vesicles (EVs), which are membrane-derived vesicles released by cells, and play an important role in cell–cell communication [113–117]. Like small unilamellar vesicle (SUV)-type liposomes, exosome-inspired nanovesicles are vesicular structures, made up of one lipid bilayer, which have a typical size ranging from 30 to 150 nm. The significant difference between SUV-liposomes and exosomes is the complicated surface structure of exosomes, with the high specificity of membrane proteins. At the same time, SUV-liposomes don’t have proteins on the lipid bilayers. Exosomes mediate intercellular crosstalk by transferring cargos, such as proteins, RNAs, DNAs, lipids, etc., to neighboring or distant cells. It also displays specific organotropic behaviour, biocompatibility, ability to communicate across biological barriers, and less immunogenicity [118–121]. Hence, exosomes have gained trending interest as a class of nano-drug delivery platforms in the past two decades [122–124]. Exosomes with modification can be acquired by modifying the progenitor cells, and isolated by ultracentrifugation, followed by further purification [113] (Fig. 3a). However, the low production and yield, composition complexity, and low drug loading efficacy inhibit its clinical translation [115]. Recently, new approaches to construct exosome-inspired nanovesicles, such as exosome mimetics and exosome mimetic hybrids, have been reported, which improved the yield and drug loading efficacy [114, 116], while maintaining their major characteristics. Exosome mimetics can be prepared by extruding cells. Exosome mimetic hybrids can be prepared by hydrating the lipid film with exosome/cell buffers (Fig. 3a). The prepared nanovesicles are also nano-sized particles (Fig. 3b). Though no exosome-based therapeutic has been approved by FDA, there are some exosome-based therapeutics have gone into clinical trials for antitumor vaccine and therapeutics. Some are in phases II–III [116]. The resource of exosomes could be genetically modified DCs, plants, tumor cells, etc.. Lu et al. [116] and Antimisiaris et al. [114] have made good reviews about exosomes and exosome-inspired vesicles as delivery systems. This part will discuss exosomes and exosome-inspired nanovesicles associated with ICB.

### Exosome mediated ICBs

As we mentioned above, ICB faces challenges such as less efficiency in tumor penetrating, systemic toxicity, etc., so people have tried to combine ICB with nanomedicines. Compared with liposomes or other nanomedicines, naturally secreted, cell-derived membranous structured exosomes have low reticuloendothelial system clearance, low immunogenicity, homing ability, and the ability to cross the blood–brain barrier and deeper tissue penetration [118, 119]. Therefore, the combination of ICB and exosomes would bring some new insights into the treatment of cancer.

DC-derived exosomes have shown the capability of augmenting antitumor CD4^+^ and CD8^+^ T cell responses, but the immunosuppressive environment limited their efficacy. Phung et al. [59] designed exosomes from ovalbumin (OVA) (antigen)-pulsed, activated DCs, and modified them with anti-CTLA-4 antibody, exosome (EXO)-OVA-mAb to synergize cancer vaccination with ICB against tumor. EXO-OVA-mAb induced strong T cell activation and proliferation in vitro, and fast migration to tumor-draining lymph nodes post subcutaneous administration in vivo. Increased migration of CD4^+^, CD8^+^ T cells, and cytotoxic T lymphocytes/Treg ratio at the tumor site was observed, and inhibited tumor progression.

Though the drug delivery system could reduce the proteolytic cleavage of ICB antibodies, improve their pharmacokinetics, and mitigate their off-target toxicity, the process of loading ICB into the delivery system, the production and storage of ICB are still challenging and costly. To solve these, Chen et al. [6] constructed a PD-L1 knockout MDA-MB-231 cell line which in the meantime overexpresses high-affinity variant human PD-1 protein (havPD-1). EVs derived from this cell line were then loaded with Senaparib (a poly ADP ribose polymerase 1/2 inhibitor, block poly ADP ribose polymerase enzyme that could stop cancer cells from repairing and allow them to die) to investigate its therapeutic efficacy in a xenograft tumor model. They chose this cell line deliberately to investigate the PD-1 immune checkpoints inhibiting. The EVs derived from MDA-MB-231 cells naturally possess the breast tumor-homing effect, which facilitates tumor targeting. They reported that havPD-1 EVs could retard the cleavage of havPD-1 by limiting its access to protease, and rapidly recognize and bind to PD-L1 expressing cancer cells. Monotherapy using havPD-1 EVs showed significant tumor growth inhibition similar to atezolizumab, and combination therapy using Senaparib-loaded havPD-1 EVs showed reduced tumor volume compared to monotherapy using low dose free Senaparib, havPD-1 EVs, or the simple mixture of Senaparib and havPD-1 EVs. This research enabled the continuous harvesting of EVs from stable engineered donor cells while having a significant tumor inhibition effect.

As a nano-drug carrier, exosomes could encapsulate multiple drugs inside, as well as antibody/ICB. Fan et al. [125] modified exosomes derived from human umbilical vein endothelial cells with anti-PD-L1 and anti-CD40 antibodies, loaded with immune drugs 2ʹ-3ʹ-cyclic guanosine monophosphate-adenosine monophosphate. Anti-PD-L1 was linked to exosome with a responsive peptide that will be cleaved in the presence of MMP-2, and the cleaved anti-PD-L1 could bind to the PD-L1 receptor on tumor cells to block the immune checkpoint. Anti-CD40 will lead to the exosome being uptaken by DCs, followed by the release of 2ʹ-3ʹ-cyclic guanosine monophosphate-adenosine monophosphate, then eventually, the production of type I IFN and proinflammatory cytokines.

In addition to immune checkpoint blocking antibodies and small molecule inhibitors, siRNAs can also be encapsulated in a nano-drug delivery system to silence messenger RNA in the cytoplasm and prevent the production of immunosuppressive molecules from the source. Pei et al. [126] co-loaded fibrinogen-like protein 1 (FGL1) and TGF-β siRNAs in exosomes derived from RAW264.7 cells. FGL1 was an inhibitory ligand of lymphocyte-activation gene 3, and TGF-β is an immunosuppressive cytokine in the TME. The co-loading of these two siRNAs silenced the expression of FGL1 and TGF-β, leading to the reshaping of the immunosuppressive TME. Exosomes were also modified with cyclic arginylglycylaspartic acid peptide to assist its targeting efficacy. Both in vitro and in vivo data proved its improved tumor inhibitory efficacy and anti-tumor immunity.

### Exosome-inspired nanovesicles mediated ICBs

In addition to the exosome-based delivery system, which is originated entirely from natural exosomes, there are many exosome-inspired nanovesicles, like exosome mimetics or hybrids, that have both biogenetic materials, for example, cell membranes and synthetic materials, like lipids. These exosome-inspired nanovesicles have more flexible preparation, and improved drug loading while maintaining the main characteristics of exosomes, such as organotropic behaviour and biocompatibility, which combine the benefits of both synthetic nanoparticles and exosomes. For example, prostate cancer, as the second-leading cancer in men, has two well-known markers, prostate-specific antigen (PSA) and prostate-specific membrane antigen (PSMA). PSA has been utilized to develop PSA cleavable prodrugs, and the latter has been used as a targeting site. Peptides that target PSMA can be transfected and expressed on cells, and these cells can be further used to prepare nanovesicles. In this case, Severic et al. [127] transfected U937 cells with anti-PSMA peptide, and prepared exosome mimetics by extruding the cells. In addition to targeting properties, these exosome mimetics are easier to prepare and purify compared to exosomes, and have a high nanovesicle yield. Ma et al. [122] reported bioinspired hybrids in the same group, using anti-PSMA expressing U937 cells and lipids. This allowed a higher encapsulation of PSA cleavable prodrug, DOX-PSA, while maintaining the PSMA targeting effect. As these exosome-inspired nanovesicles could be easily functionalized with a higher yield, it also attracts many interests, including combining it with ICB therapy.

As we know, there are many immune checkpoints, and some of them are co-expressed [65]. For example, the blockade of CD73, a checkpoint associated with adenosine metabolism that suppresses anti-tumor immune responses, can enhance the therapeutic efficacy of anti-CTLA-4 and anti-PD-1 [128]. Zhou et al. [129] prepared exosome-mimetic nanovesicles (EMVs) from macrophages (RAW264.7) and modified them with anti-PD-L1 antibodies. As the CD73-adenosine pathway plays an immunosuppressive role, and its expression may be increased in the treatment with anti-PD-L1 (EMVs-aPD-L1), they also loaded AB680, which is a CD73 inhibitor in the EMVs (AB680@EMVs-aPD-L1). AB680@EMVs-aPD-L1 treatment showed significantly improved effective T cells activation, TNF-α, IFN-γ, and IL-2 concentration in tumor tissues compared to a single treatment, either free or with EMVs. Though AB680@EMVs-aPD-L1 didn’t show a significantly better tumor inhibition effect than free AB680 + aPD-L1, it had a longer median survival (Fig. 3c) and CD8^+^/CD4^+^ ratio in tumor tissues (Fig. 3d).

Based on its function, many kinds of cells can be used to prepare nanovesicles as a delivery system. Platelets are cells that react to bleeding from blood vessel injury, and in the surgery of solid tumor removal, the wound will attract platelet accumulation. Hence, platelets could be an ideal delivery platform to eradicate residual tumor cells post tumor removal. However, platelets’ non-expendable character limited their clinical use [130]. Since platelets can be produced from megakaryocytes in vitro, Zhang et al. [130] genetically engineered murine megakaryocytes to express murine PD-1 stably, and produce PD-1 presenting mature platelets. Furthermore, the PD-1 presenting platelets were also encapsulated with cyclophosphamide, which could deplete the Tregs in TME. In their study, PD-1 presenting platelets could effectively delay the tumor growth in the B16F10 melanoma incomplete-tumor-resection model compared to free platelets or PBS. In the same model, when treated with cyclophosphamide-loaded PD-1 presenting platelets, Tregs (FoxP3^+^) decreased at the tumor site and tumor infiltrating CD8^+^ T cells significantly increased. This led to successful tumor progression suppression.

## Conclusion and perspective

Though, in many cases, ICB alone treatment won’t give the best response, using liposomal delivery could effectively combine ICB with chemotherapy, PTT/PDT, ROS, pH, enzyme response, and these could greatly enhance ICB’s therapeutic efficacy. Moreover, sometimes the co-inhibitory signals are not acting alone, dual or more ICB therapies are needed simultaneously. Using liposomal delivery could decrease the reduced synergistic effect caused by different pharmacokinetics of different ICB. With the development of nanotechnology, more and more nano-platforms for drug delivery were investigated besides liposomes. Exosomes, with their biocompatibility, specific organotropic behaviour, ability to communicate across biological barriers, and less immunogenicity have drawn more and more attention in the past two decades. Cells can be genetically modified, so the exosomes and cell-derived nanovesicles could inherit the modified peptides and receptors. Even ICB could be limited to their surface, minimizing the redundant procedures for modification as needed for other nanoparticles. However, the complexity of exosomes made clinical translation challenging. It is important to understand their composition and decisive components for their biocompatibility, specific organotropic behaviour, and ability to communicate across biological barriers.

Although a few ICBs were investigated with liposomal delivery, there is still a lot to be explored and improved. Most of the current liposomal delivery-associated ICBs focused on PD-1/PD-L1, the others are overlooked by researchers. Though researchers claim that using liposomal nanomedicine could improve ICBs’ tumor accumulation and reduce off-target toxicity, a systemic study comparing free ICBs and liposomal nanomedicine mediated ICBs is still missing, and it is very important to have this investigated. Also, most of the studies about liposomal nanomedicine mediated ICBs are combined with chemotherapy which will also lead to enhanced immunotherapeutic efficacy. Alimohammadi et al. [11] compared free anti-CTLA-4 to liposomal anti-CTLA-4, and liposomal anti-CTLA-4 showed higher tumor infiltrated lymphocytes. However, the direct comparison is very limited. How much of the improved tumor-infiltrating lymphocyte was caused by liposomal nanomedicine still needs more investigation. Additionally, most of the ICB antibodies are IgG variants that have relatively long half-life due to neonatal Fc receptor recycling [131], around 25 d for nivolumab and 15 d for ipilimumab [131–133], and this may increase irAEs [134]. However, the missing pharmacokinetic study for liposomal nanomedicine encourages more investigation.

Finally, we may give several comments related to the liposomal drug delivery system associated with immune checkpoint inhibition for cancer therapy. These include: (1) Most of the liposomes reported were PEGylated to achieve a prolonged circulation time in blood and ensure their high tumor accumulation. Nevertheless, repeat administration of PEGylated liposomes can induce rapid elimination (so-called the accelerated blood clearance, ABC phenomenon) involves the production of anti-PEG antibodies and elicit a strong immune response. Zwitterionic polymers, which have stronger surface hydration than PEG [135], could be used as the stabilizer for liposomes to solve the immunological issue, which has been shown in other nanocarrier systems. Surface modification of the liposome with antibodies will also affect its stability, so systemic studies are also needed. (2) EPR effect, the basic idea behind the liposomal drug delivery system is more effective in small animal tumor models than in human tumors. Only 14% of the phase III trials succeeded due to the lack of efficacy [136], and there have been more concerns about the EPR effect as reports say that only 0.7% of injected nanoparticles reached the TME following systemic administration [137–139]. Studies to investigate EPR effects in different tumor types should also be encouraged. Then selectively choosing to combine liposomal delivery system with ICB for a certain type of patient would be more promising. (3) Having a hydrophilic core and a lipophilic bilayer makes liposomes able to encapsulate both hydrophilic (including antibodies) and hydrophobic drugs. Liposomes’ surfaces can be easily modified with various substances, which can be done either by modifying the lipid used for the liposome before the formation of liposomes, or post-modifying when the liposomes are formed. When taking advantage of this flexibility, people should always be aware that the different payloads encapsulated or modified in/to the liposomes may result in different releasing and circulating behavior, and this should be taken into consideration when designing a new liposomal delivery system. (4) Though loading drugs/antibodies into liposomes could reduce their systemic toxicity, this would also reduce their therapeutic efficacy due to the inefficient release, and the encapsulation efficiency and loading content would also affect its applicability. Data with both encapsulation efficiency and loading content would be essential information to assess its potential for further application. However, current reports mainly gave the final encapsulation efficiency or loading content without giving the selecting process which should be encouraged to be reported. (5) The stability of the liposomal delivery system which impedes its development and performance should be considered in the early formulation stage [140, 141], and this also includes preventing burst-release of payload in the biological environment before reaching the tumor site to minimize its systemic toxicity.

For future immune checkpoint inhibition mediated with liposomal nanomedicine for cancer therapy, more immune checkpoints should be investigated, and therapeutic effects should be improved by synergistic nanomedical strategies using multiple checkpoints using. Systemic studies/comparisons of liposomes encapsulated and surfaced modified with immune checkpoints, such as pharmacokinetics, and systemic toxicity, should also be done to provide a better perspective for clinical studies. Though more systemic studies are needed, liposomal nanomedicine mediated ICBs showed great potential in reducing its irAEs and improving its therapeutic efficacy. Many efforts have been made to treat cancers, and the combination of nanotechnology with immunology is one of the ways leading us closer to success. ICB, together with liposomal delivery, are getting more promising as they have shown more efficient lymphocyte tumor infiltration, nanomedicine accumulation, and no noticeable side effect in reported in vivo studies.

## Data Availability

Not applicable.

## Abbreviations

APCs: Antigen-presenting cells
CSCs: Cancer stem cells
CTLA-4: Cytotoxic T-lymphocyte-associated antigen 4
DCs: Dendritic cells
DOX: Doxorubicin
EMVs: Exosome-mimetic nanovesicles
EPR: Enhanced permeability and retention
EVs: Extracellular vesicles
FA: Folic acid
FDA: Food and Drug Administration
FGL1: Fibrinogen-like protein 1
HT: Hyperthermia
ICB: Immune checkpoint blockade
ICD: Immunogenic cell death
IDO1: Indoleamine-2.3-dioxygenase 1
IFN: Interferon
IgG: Immunoglobulin G
irAEs: Immune-related adverse events
IRI: Irinotecan
LTSL: Low temperature-sensitive liposomes
mAb: Monoclonal antibody
MMP: Matrix metalloproteinase
NEs: Neutrophils
NIR: Near-infrared
OVA: Ovalbumin
OXA: Oxaliplatin
PD-1: Programmed cell death protein 1
PD-L1: Programmed cell death ligand 1
PDT: Photodynamic therapy
PEG: Polyethylene glycol
PSA: Prostate-specific antigen
PSMA: Prostate-specific membrane antigen
PTT: Photothermal therapy
PTX: Paclitaxel
ROS: Reactive oxygen species
siRNA: Small interfering RNA
SUV: Small unilamellar vesicle
TDZ: Thioridazine
TGF-β: Transforming growth factor-β
TIM-3: T cell immunoglobulin domain and mucin domain 3
TME: Tumor microenvironment
Treg: Regulatory T

## References

[1] Sung H, Ferlay J, Siegel RL, Laversanne M, Soerjomataram I, Jemal A (2021). Global cancer statistics 2020: GLOBOCAN estimates of incidence and mortality worldwide for 36 cancers in 185 countries. CA Cancer J Clin.

[2] Jiang X, Liu G, Li Y, Pan Y (2021). Immune checkpoint: the novel target for antitumor therapy. Genes Dis.

[3] Aguilar EJ, Ricciuti B, Gainor JF, Kehl KL, Kravets S, Dahlberg S (2019). Outcomes to first-line pembrolizumab in patients with non-small-cell lung cancer and very high PD-L1 expression. Ann Oncol.

[4] Peters S, Kerr KM, Stahel R (2018). PD-1 blockade in advanced NSCLC: a focus on pembrolizumab. Cancer Treat Rev.

[5] Khoja L, Butler MO, Kang SP, Ebbinghaus S, Joshua AM (2015). Pembrolizumab. J Immunother Cancer.

[6] Chen Y, Wang L, Zheng M, Zhu C, Wang G, Xia Y (2022). Engineered extracellular vesicles for concurrent anti-PDL1 immunotherapy and chemotherapy. Bioact Mater.

[7] Deng R, Bumbaca D, Pastuskovas CV, Boswell CA, West D, Cowan KJ (2016). Preclinical pharmacokinetics, pharmacodynamics, tissue distribution, and tumor penetration of anti-PD-L1 monoclonal antibody, an immune checkpoint inhibitor. MAbs.

[8] Cruz E, Kayser V (2019). Monoclonal antibody therapy of solid tumors: clinical limitations and novel strategies to enhance treatment efficacy. Biologics.

[9] Ai L, Chen J, Yan H, He Q, Luo P, Xu Z (2020). Research status and outlook of PD-1/PD-L1 inhibitors for cancer therapy. Drug Des Devel Ther.

[10] Xiao WY, Wang Y, An HW, Hou D, Mamuti M, Wang MD (2020). Click reaction-assisted peptide immune checkpoint blockade for solid tumor treatment. ACS Appl Mater Interfaces.

[11] Alimohammadi R, Alibeigi R, Nikpoor AR, Chalbatani GM, Webster TJ, Jaafari MR (2020). Encapsulated checkpoint blocker before chemotherapy: the optimal sequence of anti-CTLA-4 and doxil combination therapy. Int J Nanomedicine.

[12] Deveuve Q, Lajoie L, Barrault B, Thibault G (2020). The proteolytic cleavage of therapeutic monoclonal antibody hinge region: more than a matter of subclass. Front Immunol.

[13] Collins LK, Chapman MS, Carter JB, Samie FH (2017). Cutaneous adverse effects of the immune checkpoint inhibitors. Curr Probl Cancer.

[14] Morgado M, Placido A, Morgado S, Roque F (2020). Management of the adverse effects of immune checkpoint inhibitors. Vaccines (Basel).

[15] Kahler KC, Hassel JC, Heinzerling L, Loquai C, Thoms KM, Ugurel S (2020). Side effect management during immune checkpoint blockade using CTLA-4 and PD-1 antibodies for metastatic melanoma—an update. J Dtsch Dermatol Ges.

[16] Calvo R (2019). Hematological side effects of immune checkpoint inhibitors: the example of immune-related thrombocytopenia. Front Pharmacol.

[17] Lombardi A, Mondelli MU (2019). Review article: immune checkpoint inhibitors and the liver, from therapeutic efficacy to side effects. Aliment Pharmacol Ther.

[18] Barrueto L, Caminero F, Cash L, Makris C, Lamichhane P, Deshmukh RR (2020). Resistance to checkpoint inhibition in cancer immunotherapy. Transl Oncol.

[19] Russell BL, Sooklal SA, Malindisa ST, Daka LJ, Ntwasa M (2021). The tumor microenvironment factors that promote resistance to immune checkpoint blockade therapy. Front Oncol.

[20] Spranger S (2016). Mechanisms of tumor escape in the context of the T-cell-inflamed and the non-T-cell-inflamed tumor microenvironment. Int Immunol.

[21] Bulbake U, Doppalapudi S, Kommineni N, Khan W (2017). Liposomal formulations in clinical use: an updated review. Pharmaceutics.

[22] Lamichhane N, Udayakumar TS, D'souza WD, Simone CB, Raghavan SR, Polf J (2018). Liposomes: clinical applications and potential for image-guided drug delivery. Molecules.

[23] Lu Y, Gao Y, Yang H, Hu Y, Li X (2022). Nanomedicine-boosting icaritin-based immunotherapy of advanced hepatocellular carcinoma. Mil Med Res.

[24] Guimaraes D, Cavaco-Paulo A, Nogueira E (2021). Design of liposomes as drug delivery system for therapeutic applications. Int J Pharm.

[25] López-Camacho A, Higuera-Ciapara I, Velázquez-Fernández JB, Beltrán-Gracia E, Vallejo-Cardona AA (2019). Nanomedicine review: clinical developments in liposomal applications. Cancer Nanotechnol.

[26] Allen TM, Cullis PR (2013). Liposomal drug delivery systems: from concept to clinical applications. Adv Drug Deliv Rev.

[27] Barenholz Y (2012). Doxil®–the first FDA-approved nano-drug: lessons learned. J Control Release.

[28] Anselmo AC, Mitragotri S (2019). Nanoparticles in the clinic: an update. Bioeng Transl Med.

[29] Maeda H, Wu J, Sawa T, Matsumura Y, Hori K (2000). Tumor vascular permeability and the EPR effect in macromolecular therapeutics: a review. J Control Release.

[30] Allen TM, Hansen CB, de Menezes DEL (1995). Pharmacokinetics of long-circulating liposomes. Adv Drug Deliver Rev.

[31] Kobayashi H, Watanabe R, Choyke PL (2013). Improving conventional enhanced permeability and retention (EPR) effects; what is the appropriate target?. Theranostics.

[32] Wu J (2021). The enhanced permeability and retention (EPR) effect: the significance of the concept and methods to enhance its application. J Pers Med.

[33] Thakkar S, Sharma D, Kalia K, Tekade RK (2020). Tumor microenvironment targeted nanotherapeutics for cancer therapy and diagnosis: a review. Acta Biomater.

[34] Wei X, Chen X, Ying M, Lu W (2014). Brain tumor-targeted drug delivery strategies. Acta Pharm Sin B.

[35] Maeda H, Bharate GY, Daruwalla J (2009). Polymeric drugs for efficient tumor-targeted drug delivery based on EPR-effect. Eur J Pharm Biopharm.

[36] Large DE, Soucy JR, Hebert J, Auguste DT (2019). Advances in receptor-mediated, tumor-targeted drug delivery. Adv Therap.

[37] Gullotti E, Yeo Y (2009). Extracellularly activated nanocarriers: a new paradigm of tumor targeted drug delivery. Mol Pharm.

[38] Qin SY, Zhang AQ, Zhang XZ (2018). Recent advances in targeted tumor chemotherapy based on smart nanomedicines. Small.

[39] He Q, Chen J, Yan J, Cai S, Xiong H, Liu Y (2020). Tumor microenvironment responsive drug delivery systems. Asian J Pharm Sci.

[40] Guo X, Cheng Y, Zhao X, Luo Y, Chen J, Yuan WE (2018). Advances in redox-responsive drug delivery systems of tumor microenvironment. J Nanobiotechnology.

[41] Jia R, Teng L, Gao L, Su T, Fu L, Qiu Z (2021). Advances in multiple stimuli-responsive drug-delivery systems for cancer therapy. Int J Nanomedicine.

[42] Shein SA, Kuznetsov II, Abakumova TO, Chelushkin PS, Melnikov PA, Korchagina AA (2016). VEGF- and VEGFR2-targeted liposomes for cisplatin delivery to glioma cells. Mol Pharm.

[43] Zhou Q, Shao S, Wang J, Xu C, Xiang J, Piao Y (2019). Enzyme-activatable polymer-drug conjugate augments tumour penetration and treatment efficacy. Nat Nanotechnol.

[44] Hou X, Zaks T, Langer R, Dong Y (2021). Lipid nanoparticles for mRNA delivery. Nat Rev Mater.

[45] Man F, Gawne PJ, de Rosales RTM (2019). Nuclear imaging of liposomal drug delivery systems: a critical review of radiolabelling methods and applications in nanomedicine. Adv Drug Deliv Rev.

[46] Kim EM, Jeong HJ (2021). Liposomes: biomedical applications. Chonnam Med J.

[47] Cheng X, Gao J, Ding Y, Lu Y, Wei Q, Cui D (2021). Multi-functional liposome: a powerful theranostic nano-platform enhancing photodynamic therapy. Adv Sci (Weinh).

[48] Xia Y, Xu C, Zhang X, Ning P, Wang Z, Tian J (2019). Liposome-based probes for molecular imaging: from basic research to the bedside. Nanoscale.

[49] Gu ZL, Da Silva CG, Van Der Maaden K, Ossendorp F, Cruz LJ (2020). Liposome-based drug delivery systems in cancer immunotherapy. Pharmaceutics.

[50] Xing C, Li H, Li RJ, Yin L, Zhang HF, Huang ZN (2021). The roles of exosomal immune checkpoint proteins in tumors. Mil Med Res.

[51] Ou W, Jiang L, Gu Y, Soe ZC, Kim BK, Gautam M (2019). Regulatory T cells tailored with pH-responsive liposomes shape an immuno-antitumor milieu against tumors. ACS Appl Mater Interfaces.

[52] Merino M, Contreras A, Casares N, Troconiz IF, Ten Hagen TL, Berraondo P (2019). A new immune-nanoplatform for promoting adaptive antitumor immune response. Nanomedicine.

[53] Li YJ, Wu JY, Hu XB, Ding T, Tang T, Xiang DX (2021). Biomimetic liposome with surface-bound elastase for enhanced tumor penetration and chemo-immumotherapy. Adv Healthc Mater.

[54] Zhao H, Zhao B, Wu L, Xiao H, Ding K, Zheng C (2019). Amplified cancer immunotherapy of a surface-engineered antigenic microparticle vaccine by synergistically modulating tumor microenvironment. ACS Nano.

[55] Li C, Qiu Q, Gao X, Yan X, Fan C, Luo X (2021). Sialic acid conjugate-modified liposomal platform modulates immunosuppressive tumor microenvironment in multiple ways for improved immune checkpoint blockade therapy. J Control Release.

[56] Huang TY, Huang GL, Zhang CY, Zhuang BW, Liu BX, Su LY (2020). Supramolecular photothermal nanomedicine mediated distant tumor inhibition via PD-1 and TIM-3 blockage. Front Chem.

[57] Cremolini C, Vitale E, Rastaldo R, Giachino C (2021). Advanced nanotechnology for enhancing immune checkpoint blockade therapy. Nanomaterials (Basel).

[58] Lahori DG, Varamini P (2021). Nanotechnology-based platforms to improve immune checkpoint blockade efficacy in cancer therapy. Future Oncol.

[59] Phung CD, Pham TT, Nguyen HT, Nguyen TT, Ou W, Jeong JH (2020). Anti-CTLA-4 antibody-functionalized dendritic cell-derived exosomes targeting tumor-draining lymph nodes for effective induction of antitumor T-cell responses. Acta Biomater.

[60] Sobhani N, Tardiel-Cyril DR, Davtyan A, Generali D, Roudi R, Li Y (2021). CTLA-4 in regulatory T cells for cancer immunotherapy. Cancers.

[61] Vaddepally RK, Kharel P, Pandey R, Garje R, Chandra AB (2020). Review of indications of FDA-approved immune checkpoint inhibitors per NCCN guidelines with the level of evidence. Cancers.

[62] Zhang Y, Du X, Liu M, Tang F, Zhang P, Ai C (2019). Hijacking antibody-induced CTLA-4 lysosomal degradation for safer and more effective cancer immunotherapy. Cell Res.

[63] Nikpoor AR, Tavakkol-Afshari J, Sadri K, Jalali SA, Jaafari MR (2017). Improved tumor accumulation and therapeutic efficacy of CTLA-4-blocking antibody using liposome-encapsulated antibody: in vitro and in vivo studies. Nanomedicine.

[64] Sharpe AH, Pauken KE (2018). The diverse functions of the PD1 inhibitory pathway. Nat Rev Immunol.

[65] Topalian SL, Taube JM, Anders RA, Pardoll DM (2016). Mechanism-driven biomarkers to guide immune checkpoint blockade in cancer therapy. Nat Rev Cancer.

[66] Wu Y, Chen W, Xu ZP, Gu W (2019). PD-L1 distribution and perspective for cancer immunotherapy-blockade, knockdown, or inhibition. Front Immunol.

[67] Merino M, Lozano T, Casares N, Lana H, Troconiz IF, Ten Hagen TLM (2021). Dual activity of PD-L1 targeted Doxorubicin immunoliposomes promoted an enhanced efficacy of the antitumor immune response in melanoma murine model. J Nanobiotechnology.

[68] He ZD, Zhang M, Wang YH, He Y, Wang HR, Chen BF (2021). Anti-PD-L1 mediating tumor-targeted codelivery of liposomal irinotecan/JQ1 for chemo-immunotherapy. Acta Pharmacol Sin.

[69] Serrano-Del Valle A, Anel A, Naval J, Marzo I (2019). Immunogenic cell death and immunotherapy of multiple myeloma. Front Cell Dev Biol.

[70] Zhou J, Wang G, Chen Y, Wang H, Hua Y, Cai Z (2019). Immunogenic cell death in cancer therapy: present and emerging inducers. J Cell Mol Med.

[71] Jiang G, Deng W, Liu Y, Wang C (2020). General mechanism of JQ1 in inhibiting various types of cancer. Mol Med Rep.

[72] Burr ML, Sparbier CE, Chan YC, Williamson JC, Woods K, Beavis PA (2017). CMTM6 maintains the expression of PD-L1 and regulates anti-tumour immunity. Nature.

[73] Yang S, Shim MK, Song S, Cho H, Choi J, Jeon SI (2022). Liposome-mediated PD-L1 multivalent binding promotes the lysosomal degradation of PD-L1 for T cell-mediated antitumor immunity. Biomaterials.

[74] Huang L, Li Y, Du Y, Zhang Y, Wang X, Ding Y (2019). Mild photothermal therapy potentiates anti-PD-L1 treatment for immunologically cold tumors via an all-in-one and all-in-control strategy. Nat Commun.

[75] Ma G, Kostevsek N, Monaco I, Ruiz A, Markelc B, Cheung CCL (2021). PD1 blockade potentiates the therapeutic efficacy of photothermally-activated and MRI-guided low temperature-sensitive magnetoliposomes. J Control Release.

[76] Yu J, He X, Wang Z, Wang Y, Liu S, Li X (2021). Combining PD-L1 inhibitors with immunogenic cell death triggered by chemo-photothermal therapy via a thermosensitive liposome system to stimulate tumor-specific immunological response. Nanoscale.

[77] Li Y, Teng X, Wang Y, Yang C, Yan X, Li J (2021). Neutrophil delivered hollow titania covered persistent luminescent nanosensitizer for ultrosound augmented chemo/immuno glioblastoma therapy. Adv Sci.

[78] Lin B, Chen H, Liang D, Lin W, Qi X, Liu H (2019). Acidic pH and high-H2O2 dual tumor microenvironment-responsive nanocatalytic graphene oxide for cancer selective therapy and recognition. ACS Appl Mater Interfaces.

[79] Boedtkjer E, Pedersen SF (2020). The acidic tumor microenvironment as a driver of cancer. Annu Rev Physiol.

[80] Feng L, Dong Z, Tao D, Zhang Y, Liu Z (2018). The acidic tumor microenvironment: a target for smart cancer nano-theranostics. Natl Sci Rev.

[81] Gu Z, Wang Q, Shi Y, Huang Y, Zhang J, Zhang X (2018). Nanotechnology-mediated immunochemotherapy combined with docetaxel and PD-L1 antibody increase therapeutic effects and decrease systemic toxicity. J Control Release.

[82] Banstola A, Poudel K, Pathak S, Shrestha P, Kim JO, Jeong JH (2021). Hypoxia-mediated ROS amplification triggers mitochondria-mediated apoptotic cell death via PD-L1/ROS-responsive, dual-targeted, drug-laden thioketal nanoparticles. ACS Appl Mater Interfaces.

[83] Daund V, Chalke S, Sherje AP, Kale PP (2021). ROS responsive mesoporous silica nanoparticles for smart drug delivery: a review. J Drug Deliver Sci Technol..

[84] Li Y, Chen M, Yao B, Lu X, Song B, Vasilatos SN (2020). Dual pH/ROS-responsive nanoplatform with deep tumor penetration and self-amplified drug release for enhancing tumor chemotherapeutic efficacy. Small.

[85] Wang Y, Yu J, Li D, Zhao L, Sun B, Wang J (2022). Paclitaxel derivative-based liposomal nanoplatform for potentiated chemo-immunotherapy. J Control Release.

[86] Brown GT, Murray GI (2015). Current mechanistic insights into the roles of matrix metalloproteinases in tumour invasion and metastasis. J Pathol.

[87] Curran S, Murray GI (1999). Matrix metalloproteinases in tumour invasion and metastasis. J Pathol.

[88] Isaacson KJ, Martin Jensen M, Subrahmanyam NB, Ghandehari H (2017). Matrix-metalloproteinases as targets for controlled delivery in cancer: an analysis of upregulation and expression. J Control Release.

[89] Zhang M, Fang Z, Zhang H, Cui M, Wang M, Liu K (2022). Reversing tumor immunosuppressive microenvironment via targeting codelivery of CpG ODNs/PD-L1 peptide antagonists to enhance the immune checkpoint blockade-based anti-tumor effect. Eur J Pharm Sci.

[90] Lang T, Liu Y, Zheng Z, Ran W, Zhai Y, Yin Q (2019). Cocktail strategy based on spatio-temporally controlled nano device improves therapy of breast cancer. Adv Mater.

[91] Batlle E, Clevers H (2017). Cancer stem cells revisited. Nat Med.

[92] Yu Z, Pestell TG, Lisanti MP, Pestell RG (2012). Cancer stem cells. Int J Biochem Cell Biol.

[93] Lu S, Yang N, He J, Gong W, Lai Z, Xie L (2019). Generation of cancer-specific cytotoxic PD-1^-^ T cells using liposome-encapsulated CRISPR/Cas system with dendritic/tumor fusion cells. J Biomed Nanotechnol.

[94] Lian S, Xie R, Ye Y, Xie X, Li S, Lu Y (2019). Simultaneous blocking of CD47 and PD-L1 increases innate and adaptive cancer immune responses and cytokine release. EBioMedicine.

[95] Barati M, Mirzavi F, Nikpoor AR, Sankian M, Namdar Ahmadabad H, Soleimani A (2021). Enhanced antitumor immune response in melanoma tumor model by anti-PD-1 small interference RNA encapsulated in nanoliposomes. Cancer Gene Ther.

[96] Pardoll DM (2012). The blockade of immune checkpoints in cancer immunotherapy. Nat Rev Cancer.

[97] Liao Q, Zhou Y, Xia L, Cao D (2021). Lipid metabolism and immune checkpoints. Adv Exp Med Biol.

[98] Mellman I, Coukos G, Dranoff G (2011). Cancer immunotherapy comes of age. Nature.

[99] Boone CE, Wang L, Gautam A, Newton IG, Steinmetz NF (2022). Combining nanomedicine and immune checkpoint therapy for cancer immunotherapy. Wiley Interdiscip Rev Nanomed Nanobiotechnol.

[100] Guo M, Han S, Liu Y, Guo W, Zhao Y, Liu F (2019). Inhibition of allogeneic islet graft rejection by VISTA-conjugated liposome. Biochem Biophys Res Commun.

[101] Torphy R, Schulick R, Zhu Y (2017). Newly emerging immune checkpoints: promises for future cancer therapy. Int J Mol Sci.

[102] Chen YL, Lin HW, Chien CL, Lai YL, Sun WZ, Chen CA (2019). BTLA blockade enhances Cancer therapy by inhibiting IL-6/IL-10-induced CD19^high^ B lymphocytes. J Immunother Cancer.

[103] Chen S, Wainwright DA, Wu JD, Wan Y, Matei DE, Zhang Y (2019). CD73: an emerging checkpoint for cancer immunotherapy. Immunotherapy.

[104] Zhou W, Guo S, Liu M, Burow ME, Wang G (2019). Targeting CXCL12/CXCR4 axis in tumor immunotherapy. Cur Med Chem.

[105] Guo F, Wang Y, Liu J, Mok SC, Xue F, Zhang W (2016). CXCL12/CXCR4: a symbiotic bridge linking cancer cells and their stromal neighbors in oncogenic communication networks. Oncogene.

[106] Shi Y, Riese DJ, Shen J (2020). The role of the CXCL12/CXCR4/CXCR7 chemokine axis in cancer. Front Pharmacol.

[107] Lu G, Qiu Y, Su X (2021). Targeting CXCL12-CXCR4 signaling enhances immune checkpoint blockade therapy against triple negative breast cancer. Eur J Pharm Sci.

[108] Hornyak L, Dobos N, Koncz G, Karanyi Z, Pall D, Szabo Z (2018). The role of indoleamine-2,3-dioxygenase in cancer development, diagnostics, and therapy. Front Immunol.

[109] Pallotta MT, Rossini S, Suvieri C, Coletti A, Orabona C, Macchiarulo A (2022). Indoleamine 2,3-dioxygenase 1 (IDO1): an up-to-date overview of an eclectic immunoregulatory enzyme. FEBS J.

[110] Tang K, Wu YH, Song Y, Yu B (2021). Indoleamine 2,3-dioxygenase 1 (IDO1) inhibitors in clinical trials for cancer immunotherapy. J Hematol Oncol.

[111] Huang Z, Wei G, Zeng Z, Huang Y, Huang L, Shen Y (2019). Enhanced cancer therapy through synergetic photodynamic/immune checkpoint blockade mediated by a liposomal conjugate comprised of porphyrin and IDO inhibitor. Theranostics.

[112] Liu D, Chen B, Mo Y, Wang Z, Qi T, Zhang Q (2019). Redox-activated porphyrin-based liposome remote-loaded with indoleamine 2,3-dioxygenase (IDO) inhibitor for synergistic photoimmunotherapy through induction of immunogenic cell death and blockage of IDO pathway. Nano Lett.

[113] Cheng L, Hill AF (2022). Therapeutically harnessing extracellular vesicles. Nat Rev Drug Discov.

[114] Antimisiaris SG, Mourtas S, Marazioti A (2018). Exosomes and exosome-inspired vesicles for targeted drug delivery. Pharmaceutics.

[115] Ozverel CS (2020). Exosome mimetic nanovesicles; are they next best alternative therapeutic approach combating cancer?. Cyprus J Med Sci.

[116] Lu M, Huang YY (2020). Bioinspired exosome-like therapeutics and delivery nanoplatforms. Biomaterials.

[117] Kenific CM, Zhang H, Lyden D (2021). An exosome pathway without an ESCRT. Cell Res.

[118] Chen L, Wang L, Zhu L, Xu Z, Liu Y, Li Z (2022). Exosomes as drug carriers in anti-cancer therapy. Front Cell Dev Biol.

[119] Chinnappan M, Srivastava A, Amreddy N, Razaq M, Pareek V, Ahmed R (2020). Exosomes as drug delivery vehicle and contributor of resistance to anticancer drugs. Cancer Lett.

[120] Zhao Y, Liu L, Sun R, Cui G, Guo S, Han S (2022). Exosomes in cancer immunoediting and immunotherapy. Asian J Pharm Sci.

[121] Zhou K, Guo S, Li F, Sun Q, Liang G (2020). Exosomal PD-L1: new insights into tumor immune escape mechanisms and therapeutic strategies. Front Cell Dev Biol.

[122] Chen H, Wang L, Zeng X, Schwarz H, Nanda HS, Peng X (2021). Exosomes, a new star for targeted delivery. Front Cell Dev Biol.

[123] Herrmann IK, Wood MJA, Fuhrmann G (2021). Extracellular vesicles as a next-generation drug delivery platform. Nat Nanotechnol.

[124] Liang Y, Duan L, Lu J, Xia J (2021). Engineering exosomes for targeted drug delivery. Theranostics.

[125] Fan Y, Zhou Y, Lu M, Si H, Li L, Tang B (2021). Responsive dual-targeting exosome as a drug carrier for combination cancer immunotherapy. Research (Wash D C).

[126] Pei X, Zhang X, Zhang L, Yuan M, Sun L, Yu F (2021). Targeted exosomes for co-delivery of siFGL1 and siTGF-β1 trigger combined cancer immunotherapy by remodeling immunosuppressive tumor microenvironment. Chem Eng J.

[127] Severic M, Ma G, Pereira SGT, Ruiz A, Cheung CCL, Al-Jamal WT (2020). Genetically-engineered anti-PSMA exosome mimetics targeting advanced prostate cancer in vitro and in vivo. J Control Release.

[128] Allard B, Pommey S, Smyth MJ, Stagg J (2013). Targeting CD73 enhances the antitumor activity of anti-PD-1 and anti-CTLA-4 mAbs. Clin Cancer Res.

[129] Zhou Q, Ding W, Qian Z, Zhu Q, Sun C, Yu Q (2021). Immunotherapy strategy targeting programmed cell death ligand 1 and CD73 with macrophage-derived mimetic nanovesicles to treat bladder cancer. Mol Pharm.

[130] Zhang X, Wang J, Chen Z, Hu Q, Wang C, Yan J (2018). Engineering PD-1-presenting platelets for cancer immunotherapy. Nano Lett.

[131] Centanni M, Moes D, Troconiz IF, Ciccolini J, Van Hasselt JGC (2019). Clinical pharmacokinetics and pharmacodynamics of immune checkpoint inhibitors. Clin Pharmacokinet.

[132] Feng Y, Masson E, Dai D, Parker SM, Berman D, Roy A (2014). Model-based clinical pharmacology profiling of ipilimumab in patients with advanced melanoma. Br J Clin Pharmacol.

[133] O'brien T, Dolan L (2022). Immune checkpoint inhibitors and timing of administration. Lancet Oncol.

[134] Shinno Y, Goto Y, Ohuchi M, Hamada A, Nokihara H, Fujiwara Y (2020). The long half-life of programmed cell death protein 1 inhibitors may increase the frequency of immune-related adverse events after subsequent EGFR tyrosine kinase inhibitor therapy. JTO Clin Res Rep.

[135] Lin W, Kampf N, Goldberg R, Driver MJ, Klein J (2019). Poly-phosphocholinated liposomes form stable superlubrication vectors. Langmuir.

[136] He H, Liu L, Morin EE, Liu M, Schwendeman A (2019). Survey of clinical translation of cancer nanomedicines-lessons learned from successes and failures. Acc Chem Res.

[137] Wilhelm S, Tavares AJ, Dai Q, Ohta S, Audet J, Dvorak HF (2016). Analysis of nanoparticle delivery to tumours. Nat Rev Mater.

[138] De Lázaro I, Mooney DJ (2020). A nanoparticle’s pathway into tumours. Nat Mater.

[139] Challenging paradigms in tumour drug delivery. Nat Mater. 2020;19(5):477. 10.1038/s41563-020-0676-x 32332992

[140] Sainaga Jyothi VGS, Bulusu R, Rao BVK, Pranothi M, Banda S, Kumar Bolla P (2022). Stability characterization for pharmaceutical liposome product development with focus on regulatory considerations: an update. Int J Pharm.

[141] Nakhaei P, Margiana R, Bokov DO, Abdelbasset WK, Jadidi Kouhbanani MA, Varma RS (2021). Liposomes: structure, biomedical applications, and stability parameters with emphasis on cholesterol. Front Bioeng Biotechnol.

